# Molecular Cloning and Characterization of Taurocyamine Kinase from *Clonorchis sinensis*: A Candidate Chemotherapeutic Target

**DOI:** 10.1371/journal.pntd.0002548

**Published:** 2013-11-21

**Authors:** Jing-ying Xiao, Ji-Yun Lee, Shinji Tokuhiro, Mitsuru Nagataki, Blanca R. Jarilla, Haruka Nomura, Tae Im Kim, Sung-Jong Hong, Takeshi Agatsuma

**Affiliations:** 1 Department of Environmental Health Sciences, Kochi Medical School, Nankoku, Kochi, Japan; 2 Department of Parasitology, Basic Medical College, Jiamusi University, Jiamusi, China; 3 Department of Medical Environmental Biology, Chung-Ang University College of Medicine, Seoul, Republic of Korea; Khon Kaen University, Thailand

## Abstract

**Background:**

Adult *Clonorchis sinensis* lives in the bile duct and causes endemic clonorchiasis in East Asian countries. Phosphagen kinases (PK) constitute a highly conserved family of enzymes, which play a role in ATP buffering in cells, and are potential targets for chemotherapeutic agents, since variants of PK are found only in invertebrate animals, including helminthic parasites. This work is conducted to characterize a PK from *C. sinensis* and to address further investigation for future drug development.

**Methology/Principal findings:**

A cDNA clone encoding a putative polypeptide of 717 amino acids was retrieved from a *C. sinensis* transcriptome. This polypeptide was homologous to taurocyamine kinase (TK) of the invertebrate animals and consisted of two contiguous domains. *C. sinensis* TK (CsTK) gene was reported and found consist of 13 exons intercalated with 12 introns. This suggested an evolutionary pathway originating from an arginine kinase gene group, and distinguished annelid TK from the general CK phylogenetic group. CsTK was found not to have a homologous counterpart in sequences analysis of its mammalian hosts from public databases. Individual domains of CsTK, as well as the whole two-domain enzyme, showed enzymatic activity and specificity toward taurocyamine substrate. Of the CsTK residues, R58, I60 and Y84 of domain 1, and H60, I63 and Y87 of domain 2 were found to participate in binding taurocyamine. CsTK expression was distributed in locomotive and reproductive organs of adult *C. sinensis*. Developmentally, CsTK was stably expressed in both the adult and metacercariae stages. Recombinant CsTK protein was found to have low sensitivity and specificity toward *C. sinensis* and platyhelminth-infected human sera on ELISA.

**Conclusion:**

CsTK is a promising anti-*C. sinensis* drug target since the enzyme is found only in the *C. sinensis* and has a substrate specificity for taurocyamine, which is different from its mammalian counterpart, creatine.

## Author Summary

The food-borne clonorchiasis imposes public health problems on inhabitants in endemic areas. Praziquantel has been employed as an efficacious anthelminthic in large-scale campaigns as well as for individual treatment of *Clonorchis sinensis* human infections. Although praziquantel continues to have good efficacy, new drug development for this parasite has been recognized as a crucial issue to be investigated intensively. *Clonorchis sinensis* adults generate energy through glycolysis, actively utilizing exogenous glucose, and produce a large amount of eggs each day. Taurocyamine kinase (CsTK) is distributed abundantly in the locomotive and reproductive organs, and is an important enzyme in energy generation and homeostasis in adult *C. sinensis*. Enzymes of the glycolytic pathway are also expressed abundantly in these organs and in tegument, implying these organs play central roles which are essential for survival and reproduction of *C. sinensis*. The TK enzymes, including CsTK, are found only among invertebrate organisms and have substrate specificity for taurocyamine, which are significantly different from phosphagen kinases of vertebrate animals. With these molecular biological, enzymatic, and evolutionary characteristics, we propose here that CsTK could be a target for development of chemotherapeutic agents against *C. sinensis* and be a biomolecular model for other human-infecting trematodes.

## Introduction

*Clonorchis sinensis* is an important food-borne trematode parasite, which causes clonorchiasis in human and mammalian animals. This parasite is endemic in China, Korea, Taiwan, and northern Vietnam. Globally, 35 million people are estimated to be infected by *C. sinensis*, with approximately 15 million of these cases being in China [1]. The highest prevalence of *C. sinensis* human infection is reported in southern and northeastern parts of China, especially in Guangdong, Guangxi, and Heilongjiang provinces [2].

In humans, clonorchiasis can provoke severe pathologic changes in the hepatobiliary tract, and was recently recognized as belonging to the group of biological carcinogenic agents that cause cholangiocarcinoma [3], [4]. The public health and economic impact of clonorchiasis is considerable and has inspired the development of vaccines and drugs in addition to other public health measures of control and eradication the parasite itself [5].

Phosphagen kinases (PKs) catalyze a transfer of high-energy gamma phosphoryl group from ATP to a guanidino group on an acceptor molecule (phosphagen+MgADP+H+M guanidine acceptor+MgATP). The phosphorylated guanidines, called as phosphagens, serve an important molecule in cellular energy homeostasis. These phosphagens provide high-energy phosphates, which are accessible for ATP generation at substrate-level phosphorylation during periods of high metabolic demands [6]. Members of this enzyme family play a key role in the interconnection between energy production and utilization in animals. Among vertebrates, phosphocreatine is the sole phosphagen, and the corresponding kinasing enzyme is creatine kinase (CK). In invertebrates, seven unique phosphagens and corresponding kinases were identified in addition to phosphocreatine [7], [8], [9]: glycocyamine kinase (GK), taurocyamine kinase (TK), lombricine kinase (LK), opheline kinase (OK), hypotaurocyamine kinase (HTK), thalessemine kinase (ThK), and arginine kinase (AK). Several studies have also described the presence of phosphagen kinases from parasites [10]. Studies on arginine kinase from the protozoa *Trypanosoma cruzi* have identified AK as a potential target for novel drug development for Chagas' disease [11], [12]. AK has also been presented in *Toxocara canis* [13], and *Ascaris suum* [14]. Recently, two-domain TKs were reported from parasitic trematodes *Paragonimus westermani* [15] and *Schistosoma japonicum* [16]. Absence of these invertebrate PKs from the mammalian hosts, including human, imply that these kinases area possible target for new candidate drug against parasites and for development of new diagnostic reagent to detect infections.

We found a cDNA clone encoding a polypeptide (CsTK) from the *C. sinensis* transcriptome database, which was homologous with the two-domain TKs as well as the PKs of other organisms. This study was performed to elucidate biomolecular functions of CsTK such as catalytic activity comparing with TKs and PKs, tissue localization, and developmental expression.

## Materials and Methods

### Ethics statement

BALB/c mice (female, 7-week-old) and rabbits (New Zealand White, male, 2.2–2.5 kg) were handled in an accredited animal facility at Chung-Ang University (Korea FDA; Unit Number 36). Approval for animal experiments was obtained from the Institutional Animal Care and Use Committee at Chung-Ang University (Permit Number: CAU-2011-0052 and CAU-2013-0005). This study was carried out in strict accordance with the recommendations provided in the Guide for the Care and Use of Laboratory Animals by the US National Institutes of Health.

### Synthesis of *C. sinensis* total cDNA

The adult worms of *C. sinensis* were recovered from infected rabbits. Total RNA was isolated from adult worms by acid guanidinium thiocyanate-phenol-chloroform extraction method [17]. Messenger RNA (mRNA) was purified from total RNA using a poly (A)^+^ isolation kit (Nippon Gene, Tokyo, Japan). Single-stranded cDNA was synthesized with Ready-To-Go You-Prime First-Strand Beads (Amersham Pharmacia Biotech, NJ, USA) with a lock-docking oligo-dT primer [18].

### Amplification of 3′-cDNA end of *C. sinensis* PK D2 domain

Polymerase chain reaction (PCR) was carried out with reaction mixture containing cDNA, 10 pmol of each primer, 2 µl of 2.5 mM of dNTPs, 1 U of Ex Taq polymerase, 2.5 µl of 10× Ex Taq buffer (TaKaRa, Tokyo, Japan). Thermal cycles were prepared as follows: initial denaturation at 94°C for 2 min, followed by 35 cycles of 94°C for 30 s, annealing at 50°C for 35 s, and extension at 72°C for 2 min, and a final extension at 72°C for 4 min. PCR was done in a thermal cycler, MyCycler (BioRad, Foster, USA). The 3′-half of the cDNA was first amplified with lock-docking oligo (dT) primer and an “universal” redundant oligonucleotide primer 5′-GT(ACGT) TGG(AG) T(ACGT) AA(TC)GA(AG) GA(AG) GA (TC) CA-3′, designed for amplification of PK [19]. PCR products were purified using GENE CLEAN Kit (Funakoshi, Tokyo, Japan).

Purified PCR product (400 bp) was ligated into pGEMT-vector (Promega, USA) and transformed into *Escherichia coli* JM109 cells. Positive clones were obtained and plasmid DNA was extracted. Nucleotide sequences were determined with an ABI PRISM 3100-Avant DNA sequencer using a BigDye Terminators v3.1 Cycle Sequencing Kit (Applied Bio-systems, Foster, CA, USA) with two T-vector-specific primers, T7 and SP6.

### PCR amplification of 5′-half and internal region of *C. sinensis* PK D1D2 cDNA

The 5′-half of the cDNA was amplified as follows: A poly (G) ^+^ tail was added to the 5′-end of the *C. sinensis* cDNA pool with terminal deoxynucleotidy transferase (Promega, Madison, WI, USA). 5′-half of cDNA of *C. sinensis* PK was amplified using oligo-dC primer (5′-GAA TTC18-3′) and PK-csR0 primer (5′-CCA AAT TAC TCG GGC AAC AA -3′) designed on the sequence of 3′-half.

To confirm a tandem connection of CsTK D1 and D2 domains, central region of the cDNA was amplified using inner specific primers that were designed on the sequences of cDNA obtained by 5′-RACE and 3′-RACE PCR until full sequence of the cDNA was obtained [20]. With the PCR products, T-vector cloning and 3′-sequence determination of *C. sinensis* PK were performed as described above.

### Expression and purification of truncated and contiguous two-domain *C. sinensis* PKs

PCRs were done in total volumes of 50 µl. The reaction mixture contained cDNA of D1D2, 10 pmol of csPKXbaI forward primer (5′-TCT AGA ATG CAG GTC GAA CCA CTG AAA TC-3′), 10 pmol of csPKPstI reverse primer (5′-CTG CAG CTA TGG CAA GGA TTT TTC AAT AGC -3′), 1 U of KOD Plus DNA polymerase (Toyobo Co., Ltd., Tokyo, Japan), 5 µl of 10× KOD Plus buffer, 5 µl of 2 mM KOD dNTPs, and 4 µl of 25 mM MgSO_4_. The amplified products were purified using QIA quick PCR purification columns (QIAGEN GmbH, Hilden, Germany).

A-tail was added to 3′-end of the purified PCR fragments (blunt-ended). A-tailing was done in a total volume of 30 µl containing purified KOD PCR product, 15 U of Gene Taq DNA polymerase (Wako Nippon Gene), 3 µl of 10× Gene Taq buffer and 1.2 µl of 5 mM dNTP. This mixture was incubated at 70°C for 30 min. The resulting product was purified, subcloned into pGEMT-vector, and sequenced as described above.

Coding region of *C. sinensis* PK cDNA of D1D2 was cloned into XbaI/PstI site of pMAL-c2 (New England Biolabs, Ipswich, MA, USA). Maltose binding protein (MBP)-*C. sinensis* PK fusion protein was expressed in *E. coli* TB1 cells by induction with 1 mM IPTG at 25°C for 24 h. The cells were resuspended and sonicated in 5× TE buffer. Soluble recombinant fusion protein was purified by affinity chromatography using amylose resin (New England Biolabs, Ipswich, MA, USA). Homogeneity of the purified recombinant enzyme was verified by SDS–PAGE and placed on ice until assayed for enzymatic activity within 12 h. MBP-tagged CsTK D1 and D2 expressed and purified as described above.

### Amplification of *C. sinensis* taurocyamine kinase gene

Using Easy-DNA Kit (Invitrogen, Carlsbad, USA), genomic DNA was isolated from a *C. sinensis* adult worm. PCR was performed with Ex Taq polymerase (TAKARA) and primers designed on the ORF. PCR conditions were as follows: initial denaturation at 94°C for 2 min, followed by 35 cycles of 94°C for 30 s, annealing at 50°C for 30 s and extension at 72°C for 3 min and a final extension at 72°C for 4 min. PCR product was purified and sequenced as described above.

### Multiple alignment and phylogenetic analysis

Using programs CLUSTAL W (http://www.ddbj.nig.ac.jp) and GENETYXMAX (ver. 6.0), multiple sequence alignment was performed. Phylogenetic analysis was done using the distance method in MEGA (ver. 5.0). For distance analyses, the Kimura 2-parameter model was used to construct the distance matrix, and the tree was inferred from this using the Neighbor-Joining (NJ) approach. Bootstrap re-sampling was performed to assess the degree of support for groupings on the tree. Accession numbers of other amino acid sequences used in the present study are shown in Table S1.

### Site-directed mutagenesis of *C. sinensis* taurocyamine kinase

The following amino acid substitutions were amplified in the template of pMAL/*C. sinensis* TK wild type (WT): R58A, I60A, Y84A and Y84R of TKD1; H61A, I63A, Y87A and Y87R of TKD2; R58A, I60A, Y84A and Y84R of TKD1D2 in D1 region; H61A, I63A, Y87A and Y87R of TKD1D2 in D2 region. Substitution was made using KOD^+^-DNA polymerase under the subsequent PCR conditions: initial denaturation at 94°C for 2 min, followed by 35 cycles of 94°C for 15 s, annealing at 60°C for 30 s and extension at 68°C for 9 min and a final extension at 72°C for 5 min. The primer sequences designed were as follows: CsPKMutD1R58Af: 5′-GCT TGC ATC CTT CCT CGC G-3′, CsPKMutD1R58Ar: 5′- CGG ATT ACG AGC ATT GTG ACT GAC-3′; CsPKMutD1I60Af: 5′-GCT CTT CCT CGC GCT TGT GAT TTG-3′, CsPKMutD1I60Ar: 5′-GCA CCG CGG ATT ACG AGC ATT GTG-3′; CsPKMutD1Y84Af: 5′-GCT CAT AAG GTG AAA GGA GAC-3′, CsPKMutD1Y84RAr: 5′-GTC TAT AAT AAC GGC GTC AAA G-3′; CsPKMutD1Y84Rf: 5′-CGA CAT AAG GTG AAA GGA GAC-3′; CsPKMutD2H61Af: 5′-GCT TCA ATC TGT CCA CGG TAC TGG-3′, CsPKMutD2H61Ar: 5′-TGG GTT GTA AGC ACC GTT ACG-3′; CsPKMutD2I63Af: 5′-GCT TGT CCA CGT ACT GGA GAA GC-3′, CsPKMutD2I63Ar: 5′-TGA ATG TGG GTT GTA AGC ACC GT-3′; CsPKMutD2Y87Af: 5′-GCT CAT GGA GTG AGT GAC CCA GCT T-3′, CsPKMutD2Y87RAr: 5′-GTC CAA AAT CAC TGC ATC CAG GTA GTC-3′; CsPKMutD2Y87Rf: 5′-CGA CAT GGA GTG AGT GAC CCA GCT T-3′. PCR products were purified by QIAquick PCR purification column (Qiagen GmbH, Hilden, Germany). After blunting and phosphorylation, the DNA was self-ligated. Expression and enzyme assay of the mutated proteins were performed as described above.

### Enzyme assays for substrate specificity of *C. sinensis* TK

Enzyme activity was measured by absorbance at a wavelength of 340 nm (with MBP, UV/Visible Spectrophotometer 4300 Pro, Amersham, Biosciences) with an NADH-linked assay at 25°C [21], [22]. The reaction mixture (total 1.0 ml) contained 0.65 ml of 100 mM Tris–HCl (pH 8), 0.05 ml of 750 mM KCl, 0.05 ml of 250 mM Mg-acetate, 0.05 ml of 25 mM phosphoenolpyruvate prepared in 100 mM imidazole/HCl (pH 7), 0.05 ml of 5 mM NADH prepared in Tris–HCl (pH 8), 0.05 ml of pyruvate kinase/lactate dehydrogenase mixture prepared in 100 mM imidazole/HCl (pH 7), 0.05 ml of an appropriate concentration of ATP prepare in 100 mM imidazole/HCl (pH 7), and 0.05 ml of recombinant enzyme. The reaction was started by adding 0.05 ml of an appropriate concentration of guanidine substrate made up in 100 mM Tris–HCl (pH 8). Initial velocity values were obtained by varying the concentration of guanidine substrate under fixed concentrations of ATP. Protein concentration was estimated from an absorbance at 280 nm (0.77 AU at 280 nm in a 1 cm cuvette corresponds to 1 mg protein/ml).

### Quantitative real-time PCR of Cs TK

To measure mRNA transcript level in developmental stages of *C. sinensis*, quantitative real-time PCR (qRT-PCR) was performed using SYBR Green I dye with LightCycler Carousel-Based System (Roche Applied Science, Indianapolis, IN, USA). cDNAs of *C. sinensis* adults and metacercariae were employed as templates of qRT-PCR. Four pairs of forward and reverse primers were designed on each 5′- and 3′-end of CsTK D1 and CsTK D2 using Oligo6 program (Figure S1 Panel A). For qRT-PCR, forward primer 5′- TTT CCA CAA TGC CAA CAA GAC -3′ and reverse primer 5′- GCT TGA ATA CCC TGG ATG AGT -3′ on 3′-end of CsTK D2 were employed, producing a 442 bp amplicon (Figure S1, Panels B and C). qRT-PCR mix was prepared as of 1× SYBR green master mix, 1 µM gene-specific primers, and 80 ng total cDNAs. Reference genes employed were β-actin, phosphoglycerate kinase, and calcyphosine [23]. Thermal cycling of qRT-PCR started with pre-incubation at 95°C for 15 min, then continued 40 cycles of 95°C for 10 sec, 48°C for 10 sec, and 72°C for 30 sec. To verify a specific amplication of target mRNA, one melting cycle was run, 65°C for 1 min, and increase at 0.1°C/sec to 95°C to dissociate double-stranded amplicons. LightCycler software 4.05 (Roche Applied Science, Penzberg, Germany) was used to analyze melting curves and to calculate C_T_ values. A Δ^CT^ of target gene was calculated by subtracting an average C_T_ of three reference genes from an average C_T_ of the target gene. The ΔΔ^CT^ of target gene was analyzed using equation ΔΔ^CT^ = (Δ^C^_T_^target^−average of Δ^C^_T_^adult^). The 2^−ΔΔCT^ shows relative gene expression level [24].

### Purification of recombinant CsTK D1 protein

To produce and purify recombinant CsTK D1, a corresponding cDNA was subcloned into pET-23-Cs28GST vector. In this format, recombinant CsTK D1 was expressed as a fusion protein Cs28GST-tagged at its N-terminus. *E. coli* BL 21(DE3) pLysS was transformed with the expression construct and induced to produce the fusion protein using IPTG at 0.1 mM for 3 hrs. The fusion protein was absorbed to glutathione sepharose 4B column (GE Healthcare, Uppsala, Sweden) and washed with PBS. Recombinant CsTK D1 was cleaved off from Cs28GST tag on bead with 10 U/ml thrombin protease (GE Healthcare, Buckinghamshire, UK) overnight, and then eluted in PBS. Residual tagged protein was eluted in 5 mM reduced glutathione/PBS.

### Production of anti-recombinant CsTK D1 mouse immune sera

Recombinant CsTK D1 was mixed with same amount of either complete or incomplete Freund adjuvant. BALB/c mice were injected peritoneally once with 40 µg antigen/200 µl of complete adjuvant mix and again with the same amount of incomplete adjuvant mix 2 weeks later. A final buster, 1.2 µl antigen in 30 µl PBS each mouse, was injected into a tail vein after 2 weeks. After 4–5 days, blood was taken and checked for antibody production toward recombinant CsTK D1 by western blotting.

### Immunoblot of native CsTK in adult *C. sinensis*

For soluble extract, *Clonorchis sinensis* adults were washed with PBS several times and homogenized in PBS/1% Triton X-100/proteases inhibitor (1× Complete Mini, EDTA-free, Roche Diagnostics). After keeping 4°C overnight, the homogenate was spun at 20,000*×g* for 60 min at 4°C and supernatant was saved as soluble extract or crude antigen of *C. sinensis*. The soluble extract, 20 µl, was deployed in 12% SDS-PAGE and transferred onto Hybond ECL membrane (GE Healthcare, Uppsala, Sweden). The blotted membrane was incubated overnight in CsTK D1-immune mouse serum at 1∶5,000 dilution in skim milk/PBS, then in the secondary antibody, alkaline phosphatase-conjugated goat anti-mouse IgG at 1∶5,000. Color was developed using BCIP/NBT substrate (Sigma Co., St. Louis, MO, USA) and stopped in water.

### Immunohistochemical staining

Adult *C. sinensis* flukes within a rabbit liver were fixed in 10% neutral formalin and processed for paraffin blocks. The sectioned flukes in paraffin ribbons were deparaffinized and rehydrated. The *C. sinensis* ribbons were incubated in CsTKD1-immune mouse sera at 1∶200 dilution for 30 min at room temperature. Then, the sections were incubated in peroxidase- and antimouse IgG antibody-conjugated dextran polymer (to the dextran backbone, about 70 enzyme molecules and 10 primary antibodies were conjugated; Dako, Glostrup, Denmark) for 30 min at room temperature. Color was developed in AEC^+^ substrate for 5 min. Normal mouse sera were used as negative control.

### ELISA

Recombinant CsTK D1 protein, 1 µg/ml in carbonated buffer, was coated on 96-well plate at 4°C overnight. The wells were washed three times with PBS containing 0.1% Tween-20 (PBS/T) and incubated with human sera at 1∶100 dilution at 37°C for 1 hr. A secondary antibody, peroxidase -conjugated anti-human IgG (MP Biomedicals, Santa Ana, CA, USA) of 1∶4000 dilution was added to the wells and incubated at 37°C for 1 hr. Color was developed with a substrate, ophenylene diamine (Sigma Co., St. Louis, MO, USA), and optical density was measured at a wavelength of 490 nm.

Human sera used were from 47 patients with clonorchiasis, 20 with opisthorchiasis viverinii, 14 with cysticercosis cellulosae, 14 with sparganosis erinacei and from 14 patients with paragonimiasis westermani. As a control group, serum samples from 28 parasite-free human subjects were employed. A cut-off line was set at an average+doubled standard deviation which was calculated with OD values of the control group.

## Results

### Identification of the *C. sinensis* TK cDNA and Bioinformatics analysis

During large-scale cDNA sequencing of an adult *C. sinensis* cDNA library, a 2,154 bp long cDNA was successfully amplified by PCR, which encoded for a polypeptide of 717 amino acids (Fig. S2) and had 5′-UTR of 52 bp and 3′-UTR of 288 bp. Molecular mass of the polypeptide was estimated to be 80,274 Da with a pI of 7.88, using ProtParam (http://www.expasy.ch/tools/protparam.html). cDNA sequence analysis showed that this polypeptide consisted of two repetitive domains (D1 and D2) homologous to known sequences of TKs (Fig. S2). D1 contained 360 amino acids with calculated molecular mass 40,573 Da and a pI of 8.09, and D2 consisted of 357 amino acids with calculated mass 39,719 Da and a pI of 7.39. This cDNA sequence was archived in GenBank under accession number JX435779.

cDNA sequence analysis using BLASTn revealed that *C. sinensis* TK D1 and D2 had 72.3% identity with *P. westermani* TK D1 and D2. Further, the peptide sequence analysis, using BLASTp, showed that CsTK D1 and D2 were homologous with the respective domain in many TKs of different species (Fig. S2). *C. sinensis* TK polypeptide shared 79.2% sequence identity with *P. westermani* TK. CsTK D1 shared 77.7% identity with *P. westermani* TK D1 and 71.7% identity with *S. mansoni* TK D1. Meanwhile, CsTK D2 shared 82.3% identity with *P. westermani* TK D2 and 67.9% identity with *S. mansoni* TK D2 (Table 1). With this sequence information, the polypeptide conceptually deduced from the *C. sinensis* cDNA clone was considered as a new member of PK of *C. sinensis*. Residues in GS region are highly conserved across animal PKs. I60 in GS region of *C. sinensis* TK was replaced by Val in all CKs. CsTK had not a mitochondrial targeting signal peptide in N-terminus (Fig. S2).

**Table 1:** Sequence identity of *C. sinensis* TK D1 and D2 domains to other phosphagen kinases*.

	*C. sinensis* TK D1 (%)	*C. sinensis* TK D2 (%)
**Trematode TKs**		
*C. sinensis* TK D1	-	51.5
*C. sinensis* TK D2	51.5	-
*P. westermani* TK D1	77.7	50.1
*P. westermani* TK D2	50.3	82.3
*S. mansoni* TK D1	71.7	60.0
*S. mansoni* TK D2	47.1	67.9
**Sipunculid HTK**		
*S. cumanense* HTK	46.6	48.3
**Molluscan AKs**		
*C. kaikoi* AKD1	43.3	42.6
*C. kaikoi* AKD2	46.7	40.6
*T. cornutus* AK	48.9	45.1
*H. madaka* AK	51.7	46.5
**Nematode AKs**		
*T. canis* AK	39.2	39.8
*A. suum* AK	41.7	42.0
**Protozoan AKs**		
*T. cruzi* AK	41.7	46.5
*T. brucei* AK	41.3	47.5
**Arthropod AKs**		
*B. malayi* AK	43.7	35.5
*L. polyphemus* AK	43.1	45.7
**Annelid PKs**		
*A. brasiliensis* TK	34.2	32.8
*R. pachyptila* TK	36.1	31.7
*E. fetida* LK	34.4	35.6
*N. diversicolor* GK	33.1	33.6
**Mammalian CK**		
*H. sapiens* MCK	33.6	34.5

* Identity was calculated using ClustalW2 (http://www.ebi.ac.uk/Tools/msa/clustalw2/).

**Figure 1:**
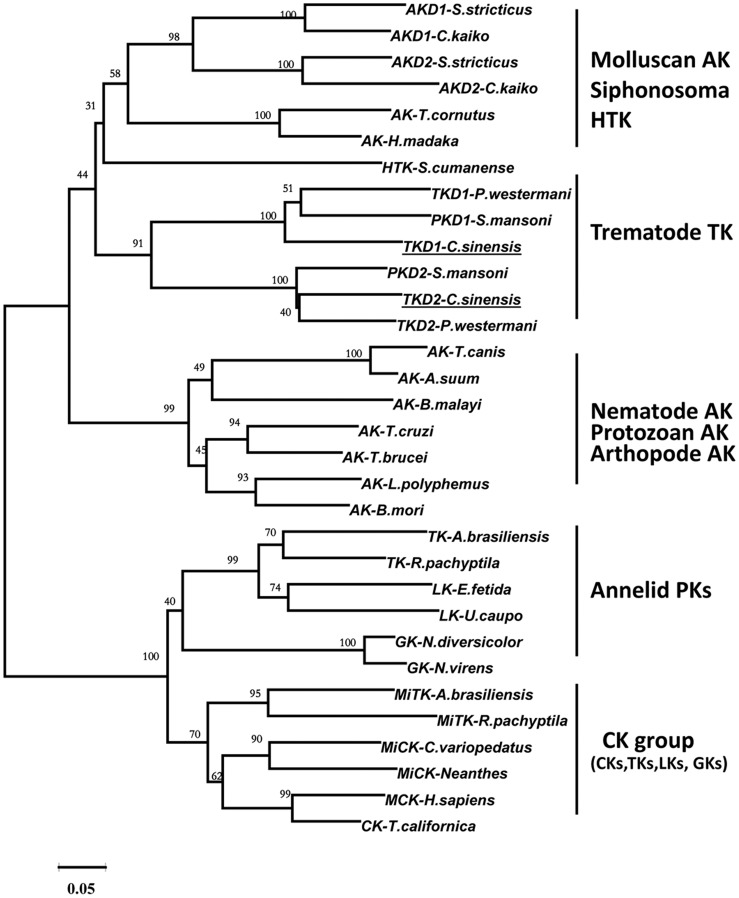
Neighbor-Joining tree for the amino acid sequences of phosphagen kinases using a program in MEGA version 5. Bootstrap values were shown at the branching point (1,000 replications). Amino acid sequences were retrieved from DDBJ and GenBank.

A phylogenetic tree, constructed using NJ algorithm (Fig. 1), indicated that PKs can be grouped into two clusters. Trematode TKs including CsTK were grouped in Cluster 1 with mulluscan AK group and nematode, protozoan, and arthropod AK group. Cluster 2 was comprised of annelid PK group and CK group of CKs, GKs, LKs, and TKs from protozoan and various insect species.

### Exon/intron organization of *C. sinensis* TK gene

**Figure 2:**
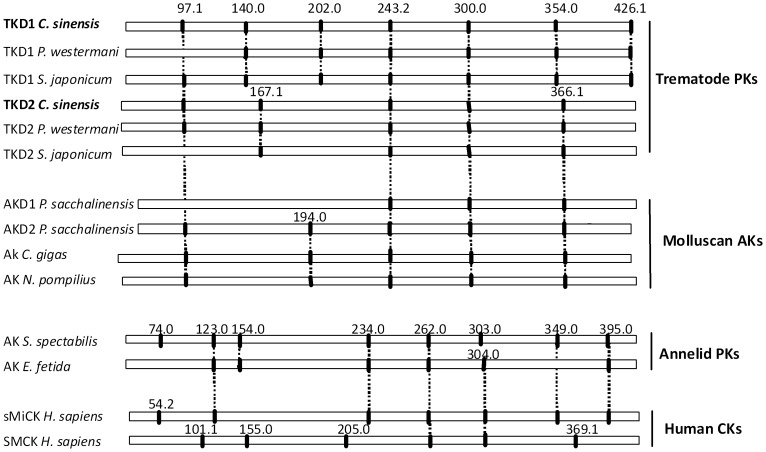
Comparison intron/exon organization of *C. sinensis* TK with other phosphagen kinases. Intron positions of *C. sinensis* TK were based on aligned amino acid sequences. Nomenclature for the intron positions was taken from Uda et al. (2006) and Jarilla (2010). Intron phase is indicated by “.0”, “.1”, or “.2” following the amino acid sequence position. Conserved introns are shown by vertical dashes.

*C. sinensis* TK gene had 13 exons and 12 introns, encoding D1 and D2 domains. Introns of *C. sinensis* TK were located at amino acid positions 97.1, 140.0, 202.0, 243.2, 300.0, 354.0, 426.1 (bridge intron) in D1, and at 97.1.1, 167.1, 243.2, 300.0, 366.1 in D2 (Fig. 2). The introns of *C. sinensis* TK began with GT and ended with AG (GT–AG pattern), except for introns at position 202.0 (GC–AG pattern) in D1. Size of the introns was variable from 115 bp to more than 4,000 bp (Table S2). Positions of *C. sinensis* TK introns were conserved between *P. westermani* TK and *S. mansoni* TK. Introns of *C. sinensis* TK 97.1, 243.2, and 300.0 shared equivalent positions with *P. westermani* TK and *S. mansoni* TK in D1 and D2 domains. Moreover, introns in *C. sinensis* TK shared additional intron positions with mollusk AKs at 97.1, 243.2, 300.0, and 366.1 (Fig. 2). The presence of these conserved intron positions supported the phylogenetic relationship of platyhelminth PKs with molluscan AKs and provided evidence for a distinct lineage of taurocyamine kinase from trematodes.

### Enzyme activity of recombinant *C. sinensis* TK

**Figure 3:**
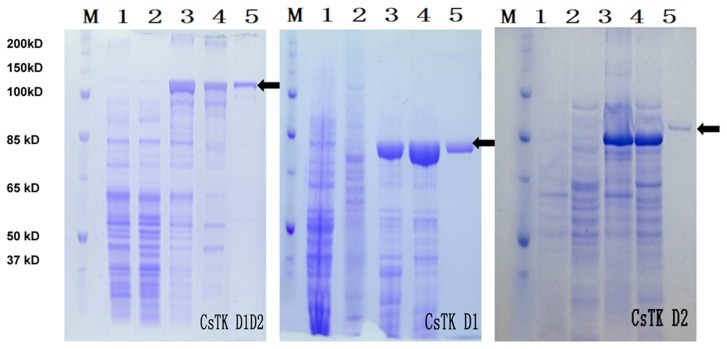
Expression and purification of recombinant *C. sinensis* TK whole, D1 and D2 domains fused to MBP in *E. coli*. M: molecular weight marker, 1, uninduced pellet; 2, uninduced supernatant; 3, IPTG-induced pellet; 4, IPTG-induced supernatant; 5, purified *C. sinensis* TK. Arrow indicates recombinant protein.

Recombinant whole and truncated variants of CsTK were successfully expressed as MBP-fusion proteins. Each set of recombinant fusion proteins was purified by affinity column chromatography to homogeneity, and appeared as a single band in SDS-PAGE CsTK D1 and D2 (truncated domain+MBP) about 80 kDa and whole CsTK 120 kDa (Fig. 3). Enzymatic activity of the recombinant CsTK was measured by NADH-linked assay for substrates taurocyamine, glycocyamine, creatine, D-arginine, and L-arginine. Whole and truncated D1 and D2 of CsTK showed enzyme activity 0.84–1.36 µmol/min·mg protein toward taurocyamine (Table 2).

**Table 2:** Enzyme activity of *C. sinensis* phosphagen kinase for various guanidine derivatives.

Substrate	PK activity (µmol/min.mg^−1^ protein)		
	D1	D2	D1D2
Taurocyamine	1.08	0.84	1.36
L-arginine	0.05	0.014	0.08
D-arginine	0.05	0.020	0.09
Creatine	0.06	0.016	0.09
Glycocyamine	0.06	0.013	0.06

### Kinetic parameter of *C. sinensis* TK

Kinetic parameter of whole CsTK K_m_^Tc^ 0.49 mM was higher than that of individual domain D1 or D2, 0.35 and 0.48 mM each, indicating that whole CsTK had lower affinity for substrate taurocyamine. CsTK D1 had stronger affinity for ATP as its *K_m_^ATP^*, 0.46 mM, was lower when compared to that of CsTK D2, 0.75 mM, and of whole CsTK, 0.78 mM. *K_cat_* value of CsTK D1, 22.59 s^−1^, was higher than that of D2, 4.50 s^−1^, and of whole CsTK, 17.78 s^−1^. Similar results were also recorded for *V_max_* and *k_cat_/K_m_^Tc^*, reflecting that CsTK D1 had more efficient catalytic activity than D2 domain or whole CsTK did. This enzymatic feature was different from that of *P. westermani* TK, of which whole *P. westermani* TK is catalytically more efficient than either of the truncated individual domains, D1 or D2 (Table 3).

**Table 3:** Comparison of *C. sinensis* recombinant TK kinetic parameters with other TKs*.

Source	Ref	*K*_m_^Tc^ (mM)	*K*_d_^Tc^ (mM)	*K*_d_^Tc^/*K*_m_^Tc^	*K*_m_^ATP^ (mM)	*K*_d_^ATP^ (mM)	*K*_d_^ATP^/*K*_m_^ATP^	*k*_cat_ (S^−1^)	*k*_cat_/*K*_m_^Tc^	*V*_max_ (umol/min. mg protein)
*C. sinensis* TK D1	Present study	0.35±0.01	2.65±0.65	7.57	0.46±0.12	2.33±0.64	5.05	22.59±0.15	54.54	33.89±2.01
*C. sinensis* TK D2	Present study	0.48±0.05	1.99±0.15	4.15	0.75±0.13	2.50±0.52	3.33	4.50±0.11	9.38	6.71±0.61
*C. sinensis* TK whole	Present study	0.49±0.02	1.92±0.43	3.92	0.78±0.07	3.46±0.70	4.44	17.78±0.98	36.26	26.68±2.11
*S.japonicum* TK D1	Tokuhiro (2012)	1.30±0.01	3.00±0.58	2.31	1.11±0.18	2.57±0.27	2.32	52.91±0.68	40.70	82.90±3.17
*S.japonicum* TK D2	Tokuhiro (2012)	0.53±0.06	1.15±0.21	2.17	1.60±0.20	3.47±0.58	2.17	15.39±0.10	29.04	28.83±1.06
*S.japonicum* TK whole	Tokuhiro (2012)	0.47±0.03	1.17±0.18	2.49	0.97±0.10	0.43±0.30	2.51	39.00±0.36	82.98	67.82±1.50
*P. westermani* TK D1	Blanca et al (2009)	0.75±0.07	4.22±1.12	5.63	0.66±0.11	3.58±0.27	5.42	24.16±1.54	32.21	40.31±2.51
*P. westermani* TK D2	Blanca et al (2009)	0.51±0.04	1.49±0.29	2.92	1.43±0.36	4.03±0.76	2.82	11.56±0.45	22.67	21.43±1.75
*P. westermani* TK whole	Blanca et al (2009)	0.57±0.10	1.95±0.43	3.42	0.98±0.16	3.37±0.70	3.44	33.44±1.01	58.67	60.01±3.01
*A. brasiliensis* TK	Uda et al (2005)	4.01±0.41	NA	NA	NA	NA	NA	9.43±0.45	2.35	28.71±1.06
*A. brasiliensis* MiTK	Uda et al (2005)	0.88±0.08	NA	NA	NA	NA	NA	14.3±1.01	16.23	17.82±1.24
*R. pachyptila* MiTK	Uda et al (2005)	2.12±0.45	NA	NA	NA	NA	NA	12.5±1.52	5.90	10.4±0.59

* Value: mean of three assays ± SD.

NA, data not available.

### Kinetic constant and catalytic efficiency of *C. sinensis* TK

As appeared in the alignment of multiple PK polypeptide sequences (Fig. S2), guanidino specificity (GS) region of CsTK had 2–3 less amino acids than that of the other known AKs, which is common feature of trematode TKs. To characterize the substrate recognition system in GS region of *C. sinensis* TK, residue substitution was introduced in CsTK (Fig. S2). Parameters of affinity, activity, and catalytic efficiency are presented in Table 4. For CsTK D1, mutations in GS region decreased its affinity for taurocyamine as evidenced by the increase of *K_m_^Tc^* values. Most significant decrease was observed in the Y84R mutant showing no enzymatic activity. Substitutions of equivalent residues in truncated D2 domain (Y84A, Y84R), whole CsTK (Y84A, Y84R in D1 and Y87A, Y87R in D2) resulted in the loss of detectable enzyme activity. Substitution of tyrosine in GS region might have affected stabilization of the closed structure of PK, suggesting that this amino acid plays an important role in taurocyamine binding. Another substitutions in D1 (I58A, R60A), and whole CsTK (I58A, R60A in D1 and H61A, I63A in D2) also decreased enzyme activity. However, mutation of equivalent position (H61A and I63A mutants) in truncated D2 increased catalytic efficiency, as evidenced by higher values of *k_cat_*, *k_cat_/K_m_^Tc^* and *V_max_*.

**Table 4:** Comparison of kinetic parameters of *C.sinensis* mutant TKs*.

Source	*K*_m_^Tc^ (mM)	*K*_d_^Tc^ (mM)	*K*_d_^Tc^/*K*_m_^Tc^	*K*_m_^ATP^ (mM)	*K*_d_^ATP^ (mM)	*k*_cat_ (s^−1^)	*k*_cat_/*K*_m_^Tc^	*V*_max_ (umol/min·mg protein)
**TK D1**	0.35±0.01	2.65±0.65	7.57	0.46±0.12	2.33±0.64	22.59±0.15	54.54	33.89±2.01
**R58A of TK D1**	0.85±0.06	2.02±0.18	2.38	0.66±0.11	1.06±0.17	33.03±1.14	38.86	30.54±1.71
**I60A of TK D1**	0.51±0.07	2.45±0.53	4.80	0.52±0.08	2.50±0.70	22.43±0.38	44.02	32.64±2.00
**Y84A of TK D1**	0.82±0.07	2.07±0.12	2.76	0.62±0.11	1.18±0.27	32.90±1.54	40.12	29.66±2.51
**Y84R of TK D1**	0	0	0	0	0	0	0	0
**TK D2**	0.48±0.05	1.99±0.15	4.15	0.75±0.13	2.50±0.52	4.50±0.11	9.38	6.71±0.61
**H61A of TK D2**	0.36±0.07	2.65±0.43	7.36	0.58±0.23	2.80±0.42	5.92±1.23	15.58	20.89±0.81
**I63A of TK D2**	0.30±0.05	2.99±0.17	9.90	0.61±0.10	2.78±0.27	6.46±0.45	21.53	26.19±2.06
**Y87A of TK D2**	3.64±0.21	3.86±0.33	1.05	NA	NA	6.45	1.77	5.19±0.24
**Y87R of TK D2**	3.01±0.25	2.41±0.17	0.80	NA	NA	0.99±0.52	0.33	1.19±0.59
**TK whole**	0.49±0.02	1.92±0.43	3.92	0.78±0.07	3.46±0.70	17.78±0.98	36.26	26.68±2.11
**R58A of D1 in TK whole**	0.45±0.19	1.72±0.43	3.82	0.76±0.12	3.43±0.70	15.00±0.25	33.33	24.15±1.11
**I60A of D1 in TK whole**	0.39±0.21	1.43±0.13	3.24	0.69±0.07	3.26±0.70	12.04±0.18	30.87	23.75±2.12
**Y84A of D1 in TK whole**	0.35±0.02	1.32±0.43	3.77	1.25±0.11	1.81±0.80	13.00±0.04	16.25	19.44±1.24
**Y84R of D1 in TK whole**	0.54±0.04	1.72±0.26	3.12	1.01±0.23	3.50±0.32	10.92±0.37	20.22	16.38±1.32
**H61A of D2 in TK whole**	0.40±0.02	1.22±0.43	3.05	0.66±0.07	3.23±0.70	12.01±0.45	30.03	23.25±1.21
**I63A of D2 in TK whole**	0.54±0.03	0.86±0.04	1.59	0.91±0.07	1.45±0.42	11.20±0.28	13.02	16.81±1.31
**Y87A of D2 in TK whole**	0.44±0.02	0.73±0.05	1.66	1.12±0.02	1.86±0.30	11.19±0.61	25.43	16.79±1.07
**Y87R of D2 in TK whole**	0.46±0.05	1.15±0.02	2.50	0.54±0.07	2.30±0.70	10.67±0.38	23.20	16.01±2.01

* Value: mean of three assays ± SD.

NA, data not available.

### Developmental expression of CsTK D1D2

**Figure 4:**
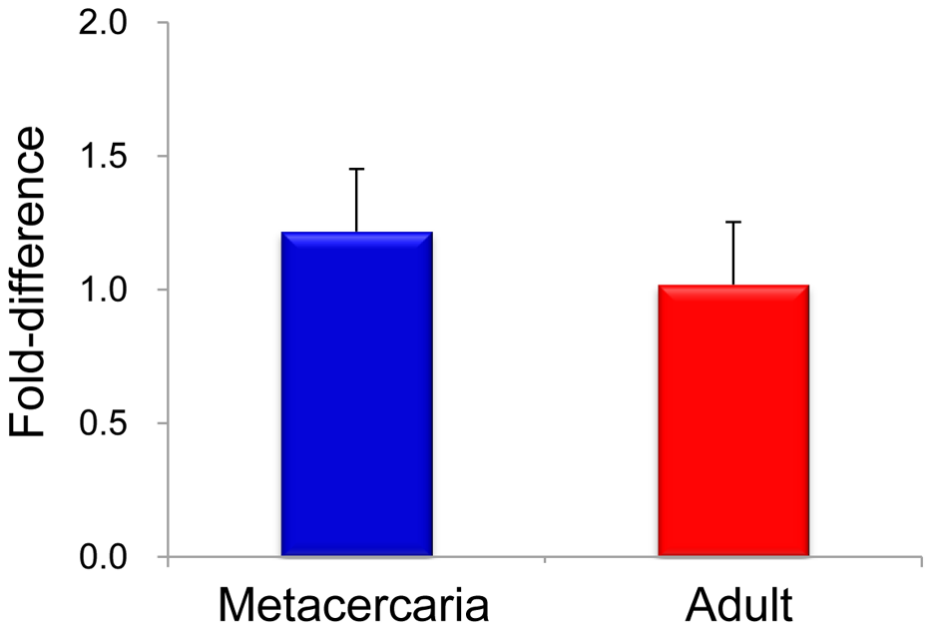
Developmental mRNA level of CsTK in *C. sinensis*. mRNA level was measured by quantitative real time PCR using a gene-specific primer pair.

To compare relative gene expression level between developmental stages by using ΔΔ^CT^ equation, three reference genes were employed such as of β-actin, phosphoglycerate kinase, and calcyphosine. CsTK D1D2 mRNA level was 1.2-fold higher in the metacercariae than in the adults of *C. sinensis* (Fig. 4)

### Recombinant Cs28GST-CsTK D1 protein

**Figure 5:**
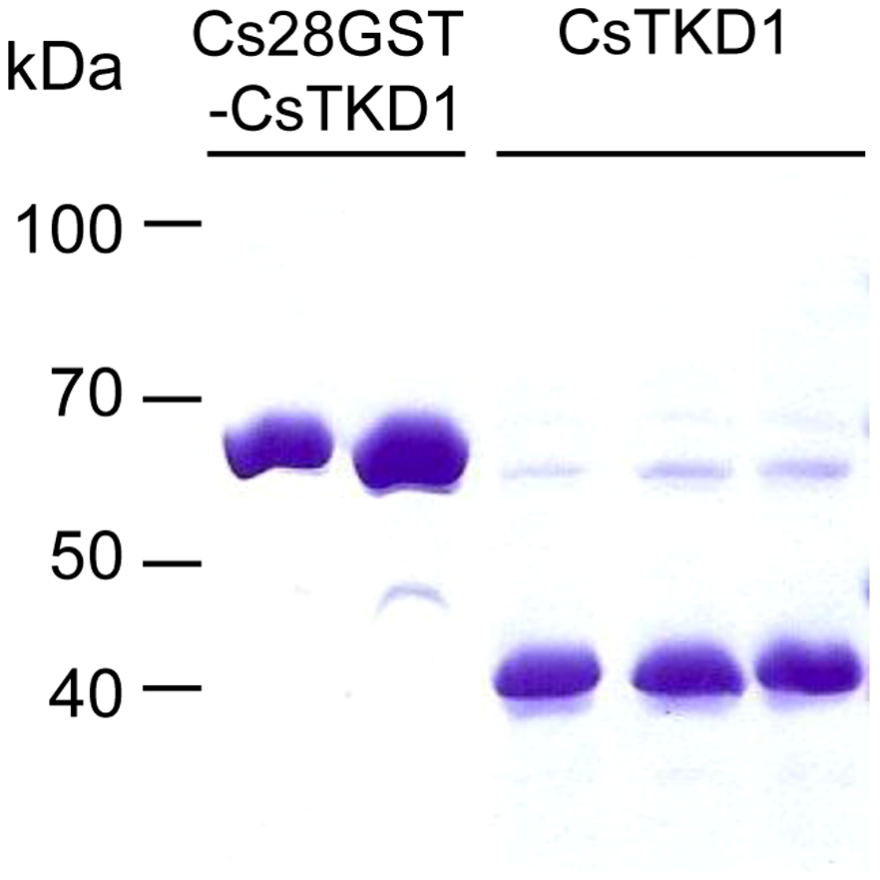
Purification of recombinant CsTKD1 by on-bead cleavage. Cs28GST-CsTKD1 was loaded to a glutathione sepharose 4B column and cleaved with thrombin. The cleaved-off CsTKD1 was eluted with PBS.

The recombinant Cs28GST-CsTK D1 fusion protein was produced as a major component and soluble form in the *E. coli* host. The recombinant CsTK D1 protein was cleaved off efficiently from the tag, Cs28GST, by thrombin treatment on bead. The cleaved CsTK D1 protein was eluted at high concentration and purity with a molecular mass of 42 kDa in SDS-PAGE gel (Fig. 5). This CsTK D1 was used for downstream experiments such as immune serum production and antigenicity tests on ELISA.

### Native CsTK protein

**Figure 6:**
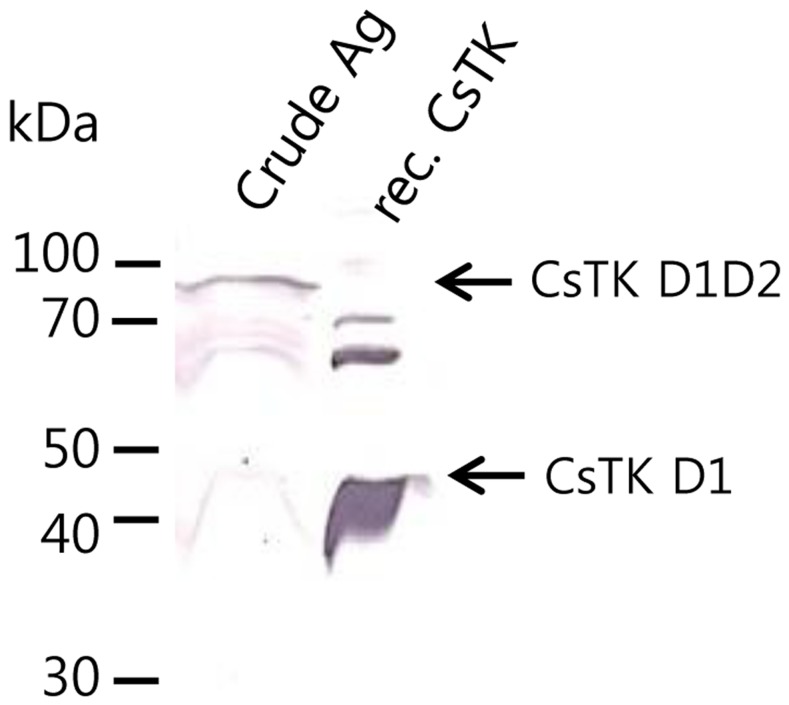
Reactivity of anti-CsTKD1 mouse immune serum to *C. sinensis* CsTK D1D2 and recombinant CsTK D1 proteins by immunoblotting.

Anti-CsTK D1 mouse immune sera reacted strongly to recombinant CsTKD1 protein. These mouse sera reacted to and detected native CsTKD1D2 protein from adult *C. sinensis* soluble extract. The native CsTKD1D2 protein was revealed as a distinctive major band of 80 kDa protein. Anti-CsTK D1 antibody was able to detect CsTK D1D2 as well as CsTK D1, since CsTK D1 and CsTK D2 are fused in tandem (Fig. 6).

### Tissue localization

**Figure 7:**
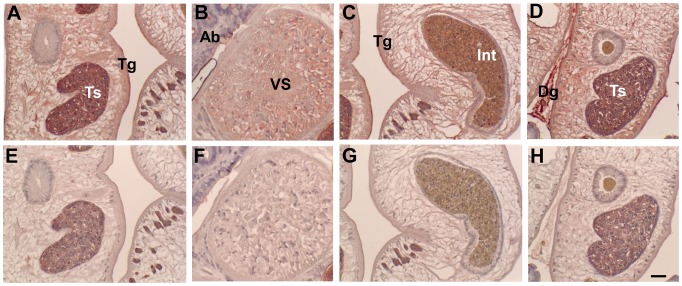
Localization of CsTK in *C. sinensis* adults by immunohistochemical staining. Upper panels A–D were stained with anti-CsTKD1 mouse sera, and lower panels E-H with normal mouse serum. Mouse anti-CsTKD1 and normal sera were used at 1∶100 dilution. Panels A and E are testis (Ts); B and F, ventral sucker (VS); C and G, intestine (Int) with content in full; D and H, Testis and dreg (Dg) between two flukes. Tg, tegument; Ab, acini of biliary epithelium. Scale bar = 50 µm.

Anti-CsTK D1 mouse immune sera were used for localization of CsTK D1D2 in the adult *C. sinensis* by immunohistochemical staining. The CsTKD1D2 was localized in tegument, oral and ventral suckers, testes, seminal vesicle and sperms, intra-uterine eggs and intestinal contents in adult *C. sinensis*. Dregs between *C. sinensis* were stained with strong positive color (Fig. 7). Acini of biliary epithelium had contents reacting positively to the immune sera.

### ELISA

**Figure 8:**
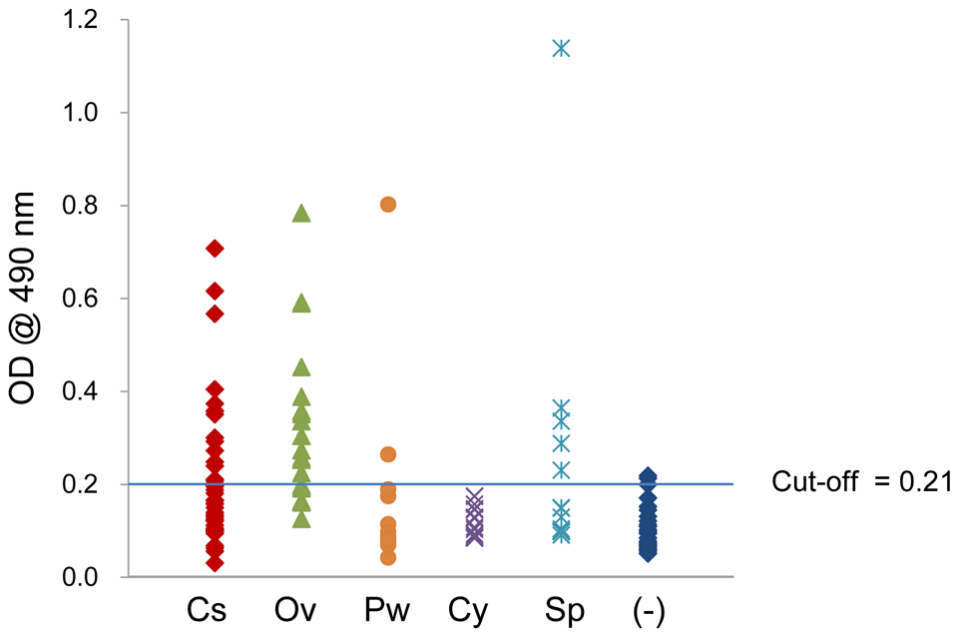
Antigenicity of recombinant CsTKD1 toward helminth-infected human sera in ELISA. Sera evaluated were of clonorchiasis (Cs), opisthorchiasis viverrini (Ov), paragonimasis westermani (Pw), cysticercosis cellulosae (Cy), sparganosis erinacei (Sp) and uninfected human controls (-). Each point is a mean of triplicate measurements.

Recombinant CsTK D1 protein was evaluated for serodiagnostic antigen toward IgG antibody in *C. sinensis*-infected human sera by ELISA. A positive cut-off value, derived from the normal control sera, was set at A_490_ = 0.21. The CsTK D1 protein gave 29.7% positive rate from sera of clonorchiasis patients. On the other hand, this positive rate was 65.0% from sera of opisthorchiasis patients, 14.3% of paragonimiasis patients, 0% from cysticercosis, 35.7% from sparganosis and 7.1% from the normal control sera (Fig. 8, Table S3).

## Discussion

Taurocyamine kinase (TK) is a member of the phosphagen kinase family, which was first isolated from the body wall muscle of polychaete lugworm, *Arenicola marina* [25]. Two types of TK, cytoplasmic TK and mitochondrial (Mi) TK were previously purified from *Arenicola brasiliensis* [26] and from deep-sea *Riftia pachyptila* [27]. As such, TKs were believed to be restricted to certain marine annelids [26]. However, *P. westermani* and *S. japonicum* TK showed activity only towards taurocyamine [15], [28]. Additionally, a two-domain phosphagen kinase with enzyme activity to taurocyamine was also reported from *S. mansoni* [29]. Thus, it was found that TK was not exclusive to marine annelids. In the present study, *C. sinensis* TK also showed exclusive activity towards taurocyamine.

In this study, we identified a cDNA clone encoding a polypeptide of 717 amino acids. The deduced amino acid sequence showed a high similarity to TKs previously reported from *P. westermani* and other helminthic parasites. It should be noted that *C. sinensis* TK had higher sequence identity with molluscan AKs than with other annelid TKs. The neighbor-joining tree revealed two major groups: CK and AK isoenzyme groups. Both domains of *C. sinensis* TK fell in AK cluster, together with *P. westermani* TK, *S. mansoni* TK, molluscan AKs and sipunculid HTK. Recombinant *C. sinensis* TK showed high enzymatic activity toward the taurocyamine substrate. With these results, it was identified for the first time that the *C. sinensis* cDNA encoded a phophagen kinase. *C. sinensis* TK was considered to be a cytoplasmic TK, since it did not have the N-terminal signal peptide of 40 residues which were the probable mitochondrial targeting sequence present in characterized mitochondrial CKs [30].

Annelid PKs had considerable catalytic efficiencies for the guanidino substrates, glycocyamine and lombricine, in addition to its original target substrate, taurocyamine [26], [27]. This was in contrast to *C. sinensis* TK, *P. westermani* TK, and *S. mansoni* TK, all of which showed exclusive enzyme activity for taurocyamine. Low degree of substrate specificity in annelid enzymes supported flexibility in substrate recognition, which might have been a driving force in the evolution of phaphagen kinases. *Eisenia* TK had obtained remarkable diversity supported by the mutation K95Y LK, dramatically changing guanidino substrate specificity from lombricine to taurocyamine [31]. Amino acid sequences of GS region of mitochondrial TK were quite different from cytoplasmic TK [32], which reflects independent evolutionary processes. Sequential difference of these two enzymes did translate to differences in enzymatic activity and substrate specificity toward the guanidino substrates, taurocyamine, lombricine, glycocyamine, and arginine [26], [27]. Number of amino acids in GS region of *C. sinensis* TK, *P. westermani* TK, and *S. mansoni* TK was smaller than those of annelid TKs (Fig. S2). Cytoplasmic TKs of *A. brasiliensis* and *R. pachyptila* were missing five residues [26], [27], and this might affect the differences in guanidino substrate specificity.

The GS region was described as a possible candidate for the guanidine recognition site, and a number of amino acid deletion in this region correlated with the size of phosphagen substrates utilized [20]. Amino acid substitutitions in the GS region resulted in a significant decrease of enzyme activity for arginine [11], [33]. However, functional properties and substrate binding mechanism of TK is not well defined yet.

To characterize substrate recognition property of *C. sinensis* TK, amino acid substitutions were put in the GS region of truncated D1 and D2 domains, and in the D1 or D2 domain of whole enzyme. The residue 140 (Fig. S2) was conserved across phosphagen kinases: even Tyr was replaced by Arg in CK, Ile in GK, His in TK, and Lys in LK [20], [26]. This residue was not directly associated with substrate binding, as revealed by the CK and AK crystal structures. However, its position was close to the guanidine substrate-binding site [33], [34], [35], and functionally, this residue determines guanidino substrate specificity [31], [36]. The residue 140 was replaced by His and Lys in cytoplasmic and mitochondrial TKs, respectively in nature. The equivalent residues in other phosphagen kinases, which correspond with the residue 95 in *Danio* CK, had the roles of distinguishing guanidino substrates and organizing the hydrogen-bond network around this position, which offered an appropriate active center for high catalytic turnover. The mode of development of this network appeared to be unique in each phosphagen kinase, reflecting the evolution of each enzyme [27]. The equivalent residue was replaced by Tyr at position 84 and 87 in *C. sinensis* TK D1 and D2 domains, and similarly in TK of *P. westermani* and *S. mansoni*. A substitution of Y84R in *C. sinensi*s TK D1 caused loss of affinity for taurocyamine. However, Y84R in D1 domain of whole CsTK still retained low enzyme activity, suggesting that two-domain structure of TK have synergistic role for enzymatic activity. Moreover, substitutions of Y84A in TK D1, Y87R and Y87A in D2, Y84A and Y84R in D1 of whole TK, Y87A and Y87R in D2 of whole TK decreased affinity for taurocyamine. It is suggested that Tyr84 in D1 domain was not a key residue for substrate recognition since replacement of this amino acid residue did not alter substrate specificity from taurocyamine to glycocyamine, but the residue is still important for taurocyamine binding.

Amino acid residues in GS region were conserved among phosphagen kinase subgroups [37]. R58 and I60 were in GS region of *C. sinensis* TK, which were key residues for catalytic activity or substrate binding among other PKs. I60 of *C. sinensis* TK was replaced by Val in all CKs, which showed low enzymatic activity for glycocyamine. The fact that the equivalent amino position of *Arenicola* MiTK V71A mutant revealed strong activity for glycocyamine suggested that the Val71 in CK might minimize its kinase activity for this substrate [38]. Substitution of H61A or I63A in CsTK D2 enhanced the enzyme's affinity for taurocyamine by about 2-fold increase. These results suggested that substitution of these two residues in the GS region affected stability of the closed structure, and that these amino acids were important for taurocyamine binding. High catalytic efficiency and strong affinity of *C. sinensis* TK toward the substrate suggested that it had a significant role in the energy metabolism for the parasite organism.

A previous study [39] reported that major AK and CK clusters could be categorized. Through phylogenetic analysis, a broad spectrum of animal PKs grouped into either an annelid-specific phosphagen kinase cluster (lombricine kinase, glycocyamine kinase, and cytoplasmic and mitochondrial TKs) or a sister-group of CKs from vertebrate and invertebrate animals. It appeared that the annelid-specific phosphagen kinases, including cytoplasmic and mitochondrial TKs, evolved from a CK-like ancestor(s) early in the divergence of the protostome metazoans. Furthermore, these results suggested that the cytoplasmic and mitochondrial isoforms of TK evolved independently [27]. It was proposed from tree topology and sequence identities that *C. sinensis* TK was in the AK subcluster with *P. westermani* TK, *S. mansoni* TK, molluscan AKs, and sipunculid HTK. Genomic organization of *C. sinensis* TK DNA, 13 exons interrupted by 12 introns, was remarkably conserved with those of *P. westermani* TK and *S. mansoni* TK. *C. sinensis* TK shared more intron positions with molluscan AKs than trematode TKs, and did not share any intron position with taurocyamine kinase from the annelid *A. brasiliensis* nor with other representative PKs belonging to the CK cluster. This result suggested that *C. sinensis* TK evolved from AK gene clad and supported the phylogeny or evolution of CsTK as shown in Fig. 1, which was different from annelid TK evolved from CK group. Phylogenetic and gene structure analyses showed that trematode TKs had evolved from a different lineage of taurocyamine kinase. Close phylogenetic relationship had been reported between flatworms and mollusks as molecular data grouped them together in the Lopotrochozoa [40]. It was hypothesized that through horizontal gene transfer and exon shuffling trematodes acquired arginine kinase from gastropod intermediate hosts, which eventually became the class of annelid taurocyamine kinases. It was hypothesized arginine kinase of the protozoa trypanosoma was a product of horizontal gene transfer from arthropods [41]. It has also been proved that *P. westermani* TK and other trematode TKs represent a distinct lineage of TKs which evolved from a molluscan AK gene rather than from a CK gene from phylogenetic and gene structure analyses [28]. 13 extron/12 intron of *C. sinensis* TK had complex gene structures compared with other *C. sinensis* genes. Phospholipid hydroperoxide glutathione peroxidase (PHGPx) and myophilin-like protein of *C. sinensis* had three and five intron, respectively [42], [43]. The results provided further insight into the evolution of taurocyamine kinase in the family of phosphagen kinases.

In our experiments, CsTK was transcribed in the metacercaria and adults of *C. sinensis*. Quantitative realtime PCR analysis revealed that the transcription level of CsTK mRNA was lower in the adult stage than in the metacercaria. This was possibly due to that the protein played an important role in growth and development of the juvenile flukes. . Moreover, immunolocalization results showed that CsTK was distributed in the tegument, testes, ventral sucker, and intestinal contents in adult *C. sinensis*. The extensive distribution and developmental stage-independent expression may imply that CsTK is a multifunctional molecule in the developmental biology of *C. sinensis*, especially in organogenesis. The tegument is an interface between parasite and its host, which was a dynamic host-interactive layer which played an important role in modulation of the host response and parasite survival [44], [45]. Moreover, tegument was one of the most active sites of energy metabolism involved with signal transduction, modulation, excretion, and osmoregulation. Previous studies have reported that proteins identified at the tegument of helminthes were suggested as important candidate antigens for drugs, immunological diagnosis, and vaccine [45], [46].

In this study, anti-CsTK D1 mouse immune sera reacted strongly to recombinant CsTKD1 protein, and these mouse sera reacted to and detected native CsTKD1D2 protein from adult *C. sinensis* soluble extract. The CsTK in the seminal vesicle, intestine and uterus of *C. sinensis* is passed out along sperms, eggs and intestinal contents into bile. The bile containing CsTK is percolated in the biliary acni and stagnated as dregs between the flukes in the biliary track, then could be presented as immunogen to the host. Through this way, the CsTK could evoke the host immune system and had decent immunogenicity.

Anti-*C.sinensis* antibody detection by enzyme-linked immunosorbent assay (ELISA) has been used for epidemiological surveys of clonorchiasis for convenience and celerity, but an ideal diagnostic and/or treatment-response assay using *C. sinensis* specific antigen or anti-body subtype could improve diagnostic sensitivity and specificity in the clinical setting. In the present study, the sensitivities of specific IgG detection and cross-reactions were measured. Recombinant CsTK D1 had low specificity and sensitivity toward sera of *C. sinensis* infected individuals.

In conclusion, a novel gene coding of *C. sinensis* TK was identified from cDNA library for the first time. CsTK gene and gene products were characterized using phylogenetic analysis, gene structure, enzyme activity, mutation, immunogenicity, antigenicity, and immunolocalization. Our current study might enhance the deduction that TK could play an important role in the growth of *C. sinensis* organism and provides clues for a promising novel candidate drug target in the control of clonorchiasis.

## Supporting Information

Figure S1 PCR amplification of CsTK cDNA from a total cDNA of *C. sinensis* adults. A, Design of PCR primers on the CsTK cDNA. B, Table of primer pairs and amplicon size. C, Amplicons electrophorated in agarose gel. Lane number as in panel B. Amplicon each in lanes 6–9 reveals the CsTK D1 and CsTK D2 cDNAs are connected in tandem.
(TIF)

Figure S2 Multiple alignment of *C. sinensis* taurocyamine kinase (TK) D1 and D2 domains with animal phosphagen kinases (PKs). The guanidine specificity (GS) region is shown in the red box. Signal peptide targeting to mitochondria is underlined. Black backgrounded residue is conserved in all PKs and gray backgrounded residue is conserved in 80% of PKs. This figure was prepared with GeneDoc (http://www.psc.edu/biomed/genedoc).
(TIF)

Table S1 Accession numbers of amino acid sequences used in present study.
(DOC)

Table S2 Intron size and the slice of boundaries sequence of *C.sinense* TK.
(DOC)

Table S3 Seroreactivity of recombinant CsTKD1 against various helminth-infected patients' and normal human sera.
(DOC)
