# Supplementary figures and images for: Molecular Cloning and Characterization of Taurocyamine Kinase from Clonorchis sinensis: A Candidate Chemotherapeutic Target
Source: PLoS Negl Trop Dis. 2013 Nov 21;7(11):e2548. doi: 10.1371/journal.pntd.0002548 (PMC3836730; doi:10.1371/journal.pntd.0002548)

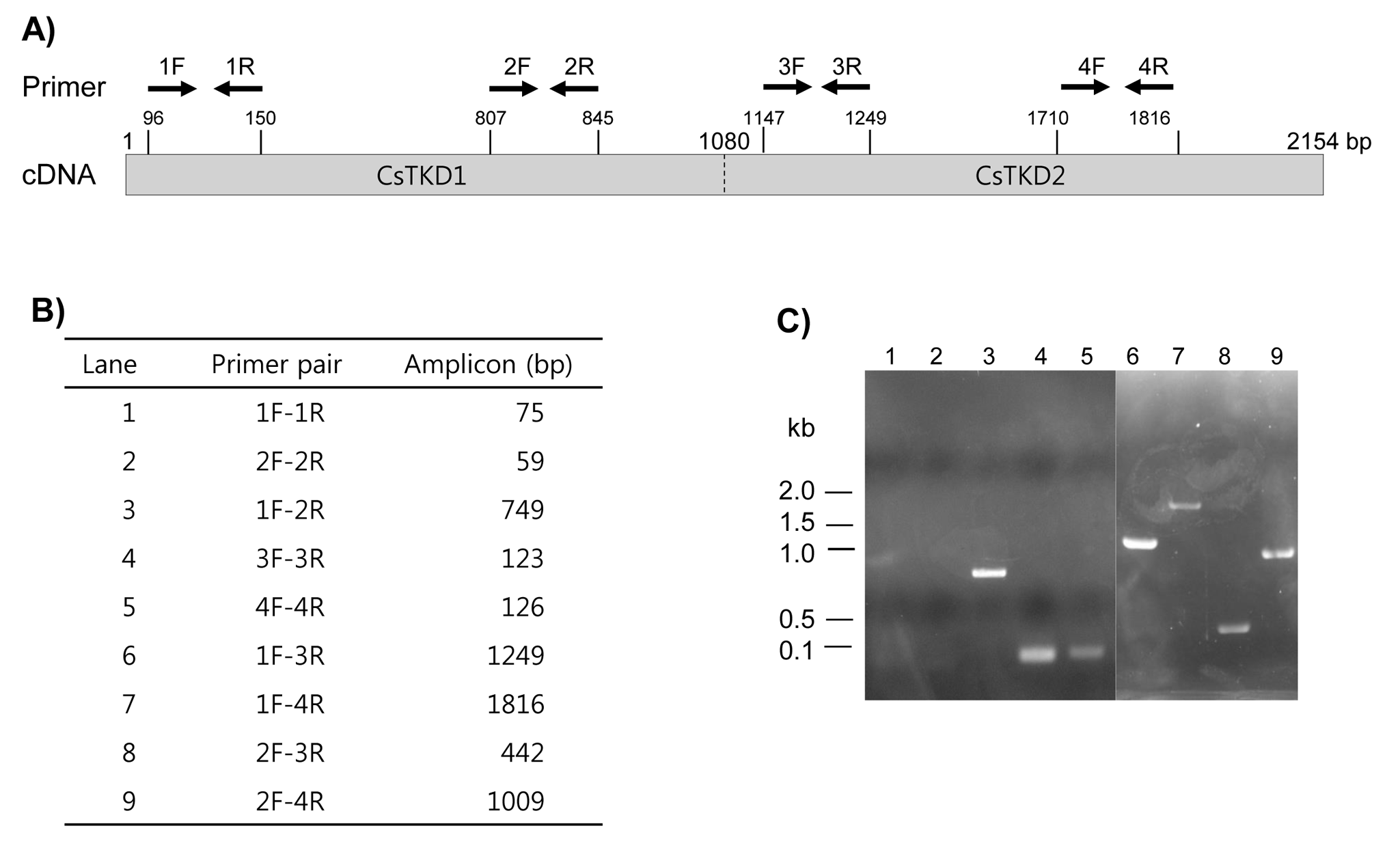

Supplement: Figure S1 — PCR amplification of CsTK cDNA from a total cDNA of C. sinensis adults. A, Design of PCR primers on the CsTK cDNA. B, Table of primer pairs and amplicon size. C, Amplicons electrophorated in agarose gel. Lane number as in panel B. Amplicon each in lanes 6–9 reveals the CsTK D1 and CsTK D2 cDNAs are connected in tandem. (TIF) [file pntd.0002548.s001.tif]

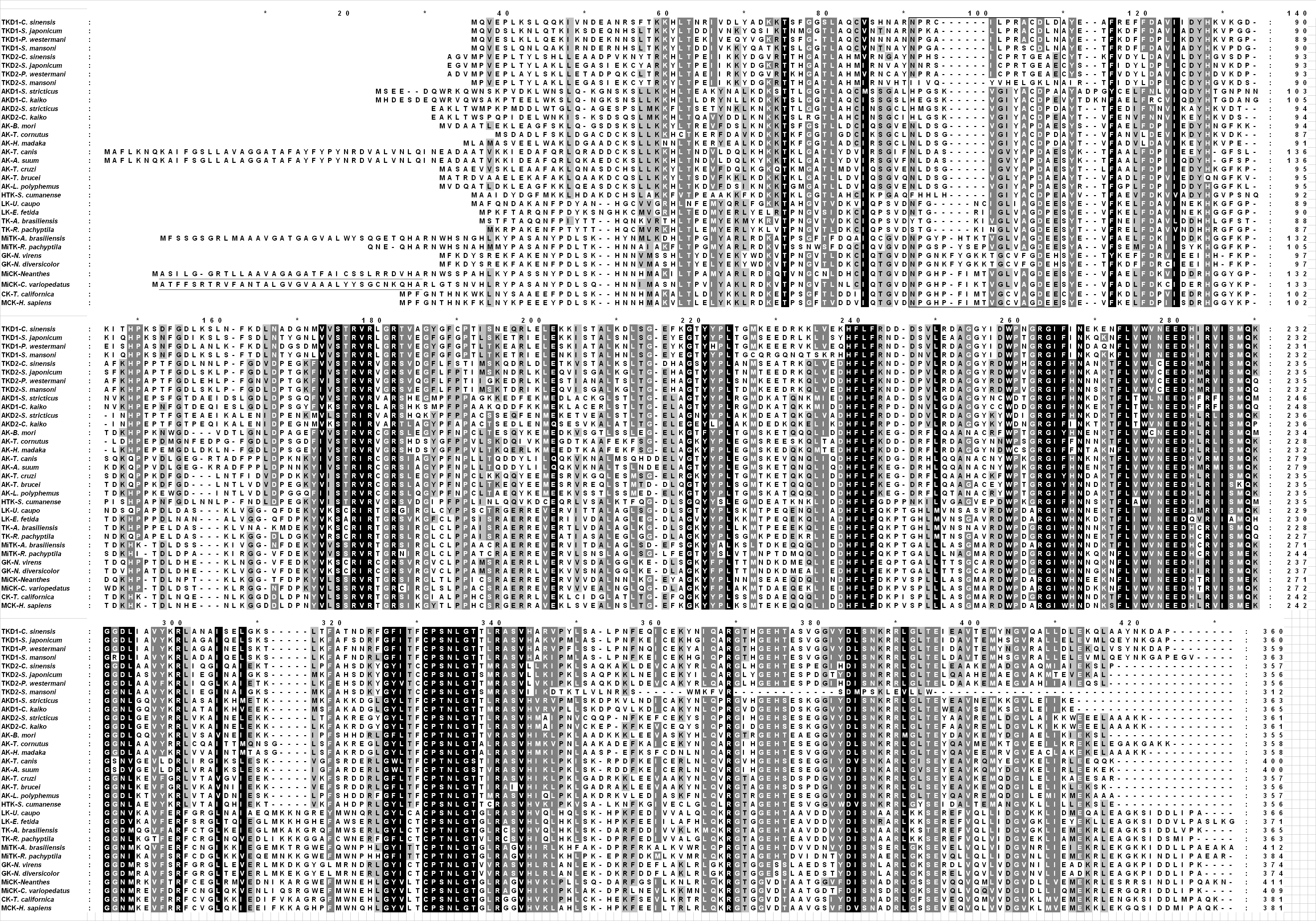

Supplement: Figure S2 — Multiple alignment of C. sinensis taurocyamine kinase (TK) D1 and D2 domains with animal phosphagen kinases (PKs). The guanidine specificity (GS) region is shown in the red box. Signal peptide targeting to mitochondria is underlined. Black backgrounded residue is conserved in all PKs and gray backgrounded residue is conserved in 80% of PKs. This figure was prepared with GeneDoc (http://www.psc.edu/biomed/genedoc). (TIF) [file pntd.0002548.s002.tif]
