# Supplementary material for: Molecular Cloning and Characterization of Taurocyamine Kinase from Clonorchis sinensis: A Candidate Chemotherapeutic Target
Source: PLoS Negl Trop Dis. 2013 Nov 21;7(11):e2548. doi: 10.1371/journal.pntd.0002548 (PMC3836730; doi:10.1371/journal.pntd.0002548)
Supplement: Table S1 — Accession numbers of amino acid sequences used in present study. (DOC) [file pntd.0002548.s003.doc]

**Table S1**. Accession numbers of amino acid sequences used in present study

| Species | Accession number |
| --- | --- |
| **Molluscan AKs** |  |
| *Solen stricticus* | AB084471.1 |
| *Calyptogena kaikoi* | [AB186413.1](http://www.ncbi.nlm.nih.gov/nuccore/AB186413.1) |
| *Turbo cornutus* | AB008011 |
| *Haliotis madaka* | P51544 |
| **Trematode TKs** |  |
| *Paragonimus westermani* TK | FJ904281 |
| *Schistosoma mansoni* TK | J05410 |
| **Sipunculid HTK** |  |
| *Siphonosoma cumanense* | AB186407 |
| **Nematode AKs** |  |
| *Toxocara canis* | EF015466 |
| *Ascaris suum* | FJ807773 |
| *Brugia malayi* | XM_001896145 |
| **Protozoan AKs** |  |
| *Trypanosoma cruzi* | AF070451 |
| *Trypanosoma brucei* | AF206293 |
| **Arthropod AKs** |  |
| *Limulus polyphemus* | P51541 |
| *Bombyx mori* | FJ013046 |
| **Annelid PKs** |  |
| *Arenicola brasiliensis* TK | BAE16473 |
| *Arenicola brasiliensis* MiTK | BAE16474 |
| *Riftia pachyptila* TK | BAE16973 |
| *Riftia pachyptila* MiTK | BAE16972 |
| *Eisenia fetida* LK | O15991 |
| *Urechis caupo* LK | AF421182 |
| *Nereis virens* GK | AAL26699 |
| *Neanthes diversicolor* GK | BAA33058 |
| *Chaetopterus variopedatus* MiCK | AAK35006 |
| *Neanthes diversicolor* MiCK | BAD34676 |
| **Chordate CKs** |  |
| *Homo sapiens* MCK | AAA96609 |
| *Torpedo californica* MCK | AAA49278 |
