# Supplementary material for: Molecular Cloning and Characterization of Taurocyamine Kinase from Clonorchis sinensis: A Candidate Chemotherapeutic Target
Source: PLoS Negl Trop Dis. 2013 Nov 21;7(11):e2548. doi: 10.1371/journal.pntd.0002548 (PMC3836730; doi:10.1371/journal.pntd.0002548)
Supplement: Table S2 — Intron size and the slice of boundaries sequence of C.sinense TK. (DOC) [file pntd.0002548.s004.doc]

**Table S2.** Intron size and the slice of boundaries sequence of *C.sinense* TK

| Intron | Sequence |
| --- | --- |
| D1 Intron1 | TCAGTCACAgtaagt (about 4000bp) ttccagATGCTCGTA |
| D1 Intron2 | TACCATAAG gtgagc (103bp) atcaagGTGAAAGGA |
| D1 Intron3 | GAGAAAAAGgcaagt (1616bp) tcaaagATTTCCACTG |
| D1 Intron4 | CGATGACAGgttagt (154bp) tttcagCGTGTTGCGC |
| D1 Intron5 | CTCGCAAACgtaagt (859bp) tttcagGCGATTTCA |
| D1 Intron6 | AATTTCGAGgtgaga (132bp) ttttagCAAATATGT |
| Bridge intron | ACGCTCCTGgtgggt (about 3500bp) ctacagCTGGTGTTA |
| D2 Intron1 | TTCGTAACGgtaagt (2954bp) tgccagTGCTTACAA |
| D2 Intron2 | ACCCTGAAGgtactt (417bp) accaagGCAAATTCG |
| D2 Intron3 | TGACGATCCgtaagc (about2800bp) taacagTGTGCTGCG |
| D2 Intron4 | CTCATCCAGgtaagt (1682bp) gttcagGGTATTCAA |
| D2 Intron5 | AAGCTCGGGgtaagc (487bp) tcctagGTCTCCACG |

Note: The intron and exon sequence are indicated by lower-case and capital, respectively
