# Supplementary material for: Molecular Cloning and Characterization of Taurocyamine Kinase from Clonorchis sinensis: A Candidate Chemotherapeutic Target
Source: PLoS Negl Trop Dis. 2013 Nov 21;7(11):e2548. doi: 10.1371/journal.pntd.0002548 (PMC3836730; doi:10.1371/journal.pntd.0002548)
Supplement: Table S3 — Seroreactivity of recombinant CsTKD1 against various helminth-infected patients' and normal human sera. (DOC) [file pntd.0002548.s005.doc]

**Table S3.** Seroreactivity of recombinant CsTKD1 against various helminth-infected patients’ and normal human sera

| Sera | No. of sera tested | Positive sera (%) |
| --- | --- | --- |
| Clonorchiasis | 47 | 14 (29.7%) |
| Opisthorchiasis | 20 | 13 (65.0%) |
| Paragonimiasis | 14 | 2 (14%) |
| Cysticercosis | 14 | 0 (0%) |
| Sparganosis | 14 | 5 (36%) |
| Normal human | 30 | 4 (7%) |
